# A case report of vaginal delivery in the second trimester of severe uterine prolapse complicated with cervical incarceration

**DOI:** 10.1097/MD.0000000000037202

**Published:** 2024-02-16

**Authors:** Xiaoyue Yang, Tongfu Feng

**Affiliations:** a Department of Ministry of Medicine, Medical College of Wuhan University of Science and Technology, Wuhan, Hubei Province, China; b Department of Gynecology of Maternal and Child Health Hospital of Hubei Province, Wuhan, Hubei Province, China.

**Keywords:** cervical incarceration, mid-term pregnancy, severe uterine prolapse, vaginal delivery, case report

## Abstract

**Background:**

Uterine prolapse is a rare complication of pregnancy, and there is still no consensus on the choice of delivery method.

**Methods:**

The patient’s reproductive history included an abortion and eutocic delivery of a girl weighing 3200 g; the current pregnancy was the third pregnancy. Her cervical region was outside the vaginal opening and was red in color, with evident enlargement (6 × 4 cm) and a broken surface. The cervical area also showed white discharge. According to her Transvaginal ultrasonography revealed a fetus in the uterine cavity at approximately 19 weeks of gestation. Gynecological examination revealed prolapse of both the anterior and posterior vaginal walls. Evaluation of the pelvic organ prolapse-Q scores showed that the patient had uterine prolapse at stage IV.

**Results:**

Vaginal delivery was performed smoothly after oral administration mifepristone and misoprostol tablets for a few days, obtaining a dead female fetus in cephalic, 25 cm in length. The cervix of the pregnant woman did not prolapse during the delivery.

**Conclusion:**

For pregnancy with uterine prolapse and cervical incarceration, transvaginal delivery is a potential treatment option. Maintenance of cervical retraction and oral mifepristone administration with misoprostol tablets is crucial during this delivery. This treatment can minimize the risk of cervical lacerations and uterine rupture, helping surgeons to complete the operation successfully.

## 1. Introduction

When a pregnancy is terminated in the second or third trimester today, there are 2 treatment choices to take into account: vaginal induction and cesarean section. More clinical choice is vaginal delivery, which has cervical dilatation issues and increases the risk of uterine rupture and cervical laceration if the cervix is not ripe. Cesarean section is the second choice. It is currently primarily utilized in those who cannot tolerate vaginal induction or who need to end a pregnancy right away due to major risks, including imminent uterine rupture and significant bleeding that can happen during induced labor.

Uterine prolapse is a condition where the uterus moves down into or beyond the vagina, due to a failure of the support by uterine and sacral ligament. It is generally quantified by clinical examination using the International Continence Society Pelvic Organ Prolapse Quantification (ICS POP-Q) system, published in 1996.^[1]^ Female pelvic organ prolapse (POP) is a common condition among older women, but it is rarely seen among women who are of reproductive age, with an incidence of 1/15,001 to 1/10,001.^[2]^ Uterine prolapse may increase the risk of complications during vaginal delivery due to the distortion of pelvic anatomy.

Cervical incarceration is a rare pregnancy complication that occurs when the cervix prolapses outside of the vaginal opening and does not naturally retract. The pregnant uterus grows and applies increasing pressure on the pelvic organs, complicating attempts at retraction. A vaginal delivery with cervical incarceration is at risk of complications including ripping cervix rupture and even uterine rupture.

In this case, vaginal delivery was successfully induced in the second trimester of severe uterine prolapse complicated with cervical incarceration. To our knowledge, such cases are rarely reported,^[3,4]^ and similar case management is still controversial,^[5–9]^ which needs more research and summary.

## 2. Case presentation

A 33-year-old woman was admitted to the gynecological department with a mass that was coming out of the vagin. She said the mass had been out for 3 weeks and had worsened in the last 3 days. The first 3 weeks, the lowest point of the mass was at the proximal part of the Hymen, and the mass can be retract after a break. During this period, the patient had no complications such as lower stomach pain, lumbosacral pain, urgency, frequent urination, vaginal discharge, or urgency and had not received any special treatment. Due to the mass has grown rapidly in the last 3 days, the patient came to the hospital for treatment.

According to her, the prolapsed mass was no longer able to return on its own, which had been accompanied by growing vaginal discharge and low back pain. In transvaginal ultrasonography (USG), a live fetus was observed, the ultrasound gestational week was 19 weeks, and there was a cystic solid mass in the fetus’ frontal region (consider encephalocele). The patient stated that she was not yet aware of her pregnancy status because she had irregular menstrual cycles. Several vaginal bleeds occurred during the pregnancy, although they were less frequent than before. She mistakenly believed she was menstruating. The reproductive history included an abortion and a eutocic delivery of a girl weighing 3200 g, the current pregnancy is the third pregnancy. Due to a malformed fetal brain, the patient and her family were insistent on ending the pregnancy.

**Figure 1.**
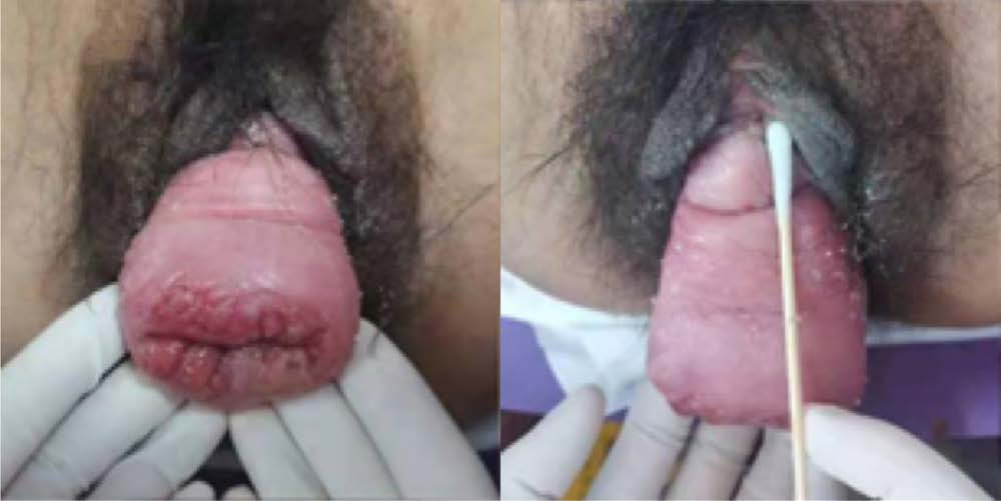
The patient’s cervical was outside the vaginal opening, and is red in color, with evident enlargement (6 × 4 cm), and a broken surface.

On examination, the abdomen was bulging, with a circumference of 68 cm. The fundus of the uterus was located 3 fingers below the umbilicus when the mass came out. Following manual cervical return, the fundus was rises to the level of the 2 fingers below the umbilicus. The patient’s cervical was outside the vaginal opening, and is red in color, with evident enlargement (6 × 4 cm), and a broken surface (Fig. 1). The cervical area also showed white discharge. Both the anterior and posterior walls of the vagina are prolapsed. the evaluation from POP-Q scores as shown in Table 1. Admission diagnosis: mid-term abortion, urinary prolapse stage IV, cervical incarceration, cervical erosion, and vaginitis.

**Table 1:** Comparison of POP-Q scores in this patients, before and after delivery.

Stage	Aa point	Ba point	C point	Ap point	Bp point	D point	TVL point	Gh point	Pb point
Before delivery (cm)	+2	+5	+8	+1.5	+1.5	−4	10	4	3
After delivery (cm)	+1	+0	+0	−2	−2	8	10	4	3

Aa midline of the anterior vaginal wall, 3 cm proximal to the external urethral meatus; Ba most distal position of any part of the upper anterior vaginal wall from the vaginal cuff or anterior vaginal fornix to point Aa. Without any prolapse, point Ba is −3 cm; C most distal edge of the cervix or the leading edge of the vaginal cuff after hysterectomy; Ap located in the midline of the posterior vaginal wall, 3 cm proximal to the hymen. The possible values range from −3 to +3; Bp the most distal position of any part of the upper posterior vaginal wall from the vaginal cuff or posterior vaginal fornix to point Ap. Bp is −3 when there is no prolapse; POP-Q pelvic organ prolapse quantitative

**Figure 2.**
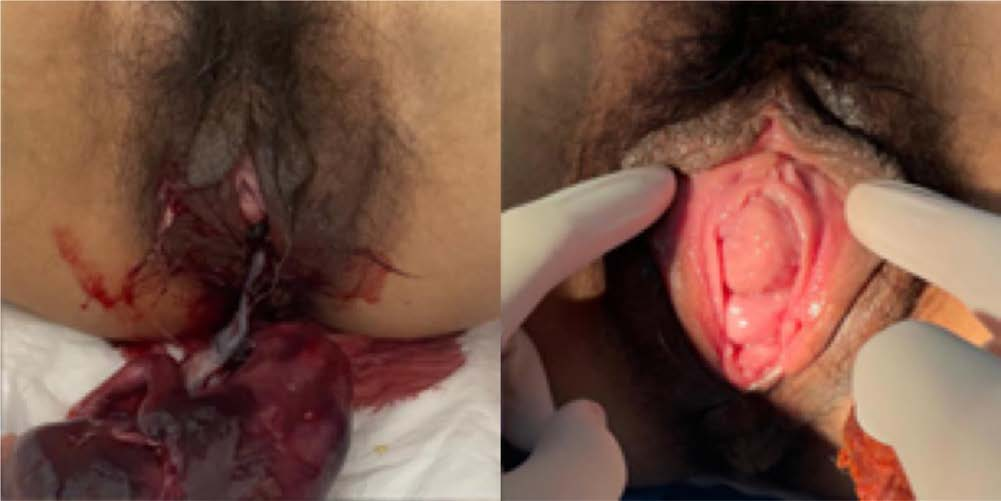
The symptoms of uterine prolapse were alleviated from stage IV to stage II.

**Figure 3.**
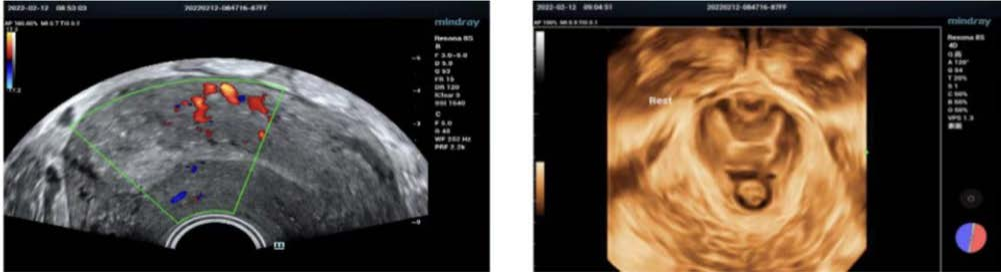
On the second postpartum day, in transvaginal USG, a 3.0 cm tissue was seen in the left side of the uterine cavity. The pelvic organ was not obvious damaged.

Due to the swollen and cracked cervix was protruding outside the vagina, the prolapse was returned manually and a daily cleaning and disinfecting was carried out to the cervix. These made the patient’s cervical edema to progressively lessen. After 2 days of oral mifepristone (60 mg/BID), the contractions gradually became more intensified following the oral dose of misoprostol tablets (600 µg) at 6 am. A vaginal delivery was carried out smoothly, obtaining a dead female fetus in cephalic, 25 cm in length. The cervix of the pregnant woman did not prolapse during delivery. On the second postpartum day, compared with the condition before the delivery, the pelvic prolapse significantly improved on the basis of the evaluation of POP-Q scores, as shown in Table 1. The symptoms of uterine prolapse were alleviated from stage IV to stage II (Fig. 2). The fundus of the uterus was located 2 fingers above the pubic symphysis. On the second postpartum day, in transvaginal USG, a 3.0 cm tissue was seen in the left side of the uterine cavity. Pelvic organ was not obvious damaged (Fig. 3). The patient refused to perform the curettage, and requested to take oral mifepristone for 3 days, and then take the ultrasound again. She was discharged from the hospital on the third day after giving birth. In order to improve the long-term prognosis, the patients were advised to adjust their living habits as far as possible and to do pelvic floor rehabilitation exercise. During 1-month follow-up examinations, the patient’s prolapse did not worsen, this received high patient satisfaction.

## 3. Discussion and conclusions

In women of childbearing age, uterine prolapse during pregnancy is less prevalent, and it is much more uncommon, especially when it is complicated with cervical incarceration. It can be difficult for a gynecologist to decide how to induce labor in cases of significant uterine prolapse and cervical incarceration during midterm pregnancy. When choosing the method of inducing labor in these patients, it’s crucial to take into account the possibility that a vaginal trial may worsen the patient’s prolapse symptoms or other serious complications, such as severe cervical lacerations. Meanwhile, we should keep in mind that a cesarean section is not always the best option. These patients have a higher risk of vaginal delivery, but it is also possible to tolerate that. Nowadays, the treatment of such cases remains controversial.^[3–9]^ More summarization and analysis are needed in an effort to better comprehend it and offer treatment guidelines. This case shows that pregnant women with prolapse of the uterus can attempt vaginal delivery under close monitoring. The results after vaginal delivery were similar to those after cesarean section, prolapse symptoms can be greatly decreased.^[6,7]^

Patients who need induction in the middle of pregnancy are at a risk because the cervix is not yet mature, the cervix is sometimes difficult to dilate, and this can cause labor to become obstructed, leading to “cervical dystocia,” cervix laceration, or even can rupture the uterus or cause the formation of a pelvic hematoma. Therefore, it is essential to administer the right medications under medical supervision in order to enhance cervix ripening and facilitate delivery.^[10]^ Between 11 and 24 weeks of gestation, the fetal skeleton is essentially developed, the uterus is congested and enlarging, and the volume of amniotic fluid starts to progressively increase. Artificial negative pressure suction can no longer be used to terminate pregnancies, and water sac surgery to induce labor is more complex and risky. The most effective method to end a pregnancy is to use mifepristone and misoprostol together. The approach is secure and reliable.^[11–13]^ There is no doubt that the patient is currently unable to use a cervical water sac. Meanwhile, if Acrinol is given intra-amniotically to stimulate uterine contractions, it is simple to create stronger uterine contractions, gradual cervical dilatation out of sync, or even cervical laceration. Therefore, the cervix was only softened, and contractions were induced with mifepristone and misoprostol tablets.

In this case, the patient was subjected to cervical incarceration. The cervix became hyperemic, edematous, and enlarged following cervical imprisonment due to blood flow restriction, making it increasingly harder to retract. With continued friction, infections, and ulcers grow, the danger of ripping the cervix is higher because the surface of the cervix is dry and cracked. Before delivery, the cervical circumstances must be improved. In this case, the patient’s cervical swelling significantly diminished only after the cervical retract into the vagina. This indicates that retracting the cervix can reduce edema and enhance cervical blood flow. To keep the cervix retracted as long as possible, patients were also told to adopt a low head and high hip position at the same time. On the other hand, cervical edema can be treated locally with a moist application of magnesium sulfate, if necessary.

Another significant danger of induced labor is the retained placenta. In this instance, oral mifepristone was chosen because, in addition to its effects on the cervix of the pregnant woman, it can also affect the patient’s endometrium, caused denaturation, necrosis, continued damage to the chorionic villi, and exfoliation of the cells in the decidua tissue, which made the patient’s placenta easier to expel. Oral mifepristone can also aid in removing part of the leftover placenta after inducing birth.

How to select the method of pregnancy termination in such cases is a challenging issue since there is no clear consensus on which is the most appropriate therapeutic option. It is important to conduct a thorough analysis of the overall scenario. Depending on the degree of uterine prolapse and cervical ripening at the time of the disease’s development, individualized treatment approaches might be taken during pregnancy. Additionally, it’s important to increase the monitoring of the labor process, pay attention to tonic contractions, safeguard the soft delivery canal, and promptly identify and address any major issues like cervix rupture.

In addition, for these pregnant women, postpartum uterine prolapse prevention and therapy should be implemented. It seems reasonable to carry out a good job of pregnant women’s knowledge propaganda about pregnancy and postpartum care, and we should optimize pregnant women’s fetal delivery techniques, strengthen the postpartum observation and care of pregnant women.

As the number of multipartially pregnant women and senior pregnant women rises, pregnancy with uterine prolapse will become an increasingly common problem. In this instance, it is clear that despite severe uterine prolapse and cervical incarceration in the second trimester of the pregnancy, vaginal delivery is still an option. The prognosis after vaginal delivery is also positive, and the symptoms of uterine prolapse do not worsen dramatically.

## Author Contributions

**Formal analysis:** Xiaoyue Yang.

**Writing—original draft:** Xiaoyue Yang.

**Writing—review & editing:** Xiaoyue Yang, Tongfu Feng.

**Funding acquisition:** Tongfu Feng.

**Project administration:** Tongfu Feng.

**Supervision:** Tongfu Feng.
